# Prostate Osteoblast-Like Cells: A Reliable Prognostic Marker of Bone Metastasis in Prostate Cancer Patients

**DOI:** 10.1155/2018/9840962

**Published:** 2018-12-09

**Authors:** Manuel Scimeca, Nicoletta Urbano, Bonfiglio Rita, Sarah Natalia Mapelli, Carlo Vittorio Catapano, Giuseppina Maria Carbone, Sara Ciuffa, Mario Tavolozza, Orazio Schillaci, Alessandro Mauriello, Elena Bonanno

**Affiliations:** ^1^Department of Biomedicine and Prevention, University of Rome “Tor Vergata”, Via Montpellier 1, Rome 00133, Italy; ^2^University San Raffaele, Via di Val Cannuta 247, 00166 Rome, Italy; ^3^Nuclear Medicine, Policlinico “Tor Vergata”, Rome, Italy; ^4^Department of Experimental Medicine and Surgery, University “Tor Vergata”, Via Montpellier 1, Rome 00133, Italy; ^5^Università della Svizzera Italiana (USI), Institute of Oncology Research (IOR), Via Vela 6, Bellinzona, Switzerland; ^6^IRCCS Neuromed, Pozzilli, IS, Italy; ^7^IRCCS Neuromed Lab. “Diagnostica Medica” and “Villa dei Platani”, Avellino, Italy

## Abstract

The main aim of this study was to investigate the putative association among the presence of prostate cancer cells, defined as prostate osteoblast-like cells (POLCs), and showing the expression of typical morphological and molecular characteristics of osteoblasts, the development of bone metastasis within 5 years of diagnosis, and the uptake of 18F-choline evaluated by PET/CT analysis. To this end, prostate biopsies (*n* = 110) were collected comprising 44 benign lesions and 66 malignant lesions. Malignant lesions were further subdivided into two groups: biopsies from patients that had clinical evidence of bone metastasis (BM+, *n* = 23) and biopsies from patients that did not have clinical evidence of bone metastasis within 5 years (BM−, *n* = 43). Paraffin serial sections were obtained from each specimen to perform histological classifications and immunohistochemical (IHC) analysis. Small fragments of tissue were used to perform ultrastructural and microanalytical investigations. IHC demonstrated the expression of markers of epithelial-to-mesenchymal transition (VIM), bone mineralization, and osteoblastic differentiation (BMP-2, PTX-3, RUNX2, RANKL, and VDR) in prostate lesions characterized by the presence of calcium-phosphate microcalcifications and high metastatic potential. Ultrastructural studies revealed the presence of prostate cancer cells with osteoblast phenotype close to microcalcifications. Noteworthy, PET/CT analysis showed higher uptake of 18F-choline in BM+ lesions with high positivity (≥300/500 cells) for RUNX2 and/or RANKL immunostaining. Although these data require further investigations about the molecular mechanisms of POLCs generation and role in bone metastasis, our study can open new and interesting prospective in the management of prostate cancer patients. The presence of POLCs along with prostate microcalcifications may become negative prognostic markers of the occurrence of bone metastases.

## 1. Introduction

Metastasis to bone is a common feature in advanced prostate cancer (PCa) patients. PCa is one of the most frequent cancer in men and represents a great public health problem, with a total of 265,000 new diagnosis every year in both Europe and United States of America [1]. Frequently, prostate cancer patients show bone osteoblastic metastatic lesions at diagnosis [1, 2]. The evidence that prostate cancer cells in patients enter the circulation in large numbers but still preferentially colonise to the bone has a number of implications. Prostate cancer cells have the ability to adhere at the main proteins of the extracellular matrix or at the bone marrow [2]. Also, the colonisation bone by prostate cancer cells suggests that metastatic cells have morphological and/or molecular characteristics that make them capable to survive in the bone [2–4].

The type of bone metastases formed in prostate cancer is a reflection of the local interaction between tumour cells and the bone remodeling system—a complex mechanism which remains to be fully characterized. Bone metastases in prostate cancer are most often osteoblastic (involving the deposition of newly formed bone), but can also be osteolytic (characterized by destruction of normal bone) or mixed. The development of either osteolytic or osteoblastic lesions results from functional interactions between tumour cells and osteoclasts or osteoblasts, respectively [5]. However, the mechanisms responsible for the formation of prostate cancer metastasis to bone are complex and certainly involve both osteoclasts and osteoblasts activity [6]. In this context, the binary classification between osteoblastic and osteolytic lesions represents two extremes of a continuum which involves dysregulation of the normal bone remodeling process and which is yet to be fully understood. A detailed characterization of the osteoblastic-osteolytic spectrum and of premetastatic tumour cells could therefore pave the way for both the identification of early markers for bone metastasis and of novel drug targets to improve quality of life of patients with advanced prostate cancer.

As concerns the origin of metastatic cells, different hypotheses have been formulated. For a long time, the main theories of the formation of bone metastases contemplated the occurrence of specific genetics change in primary cancer cells that thus acquired the ability to spread to and thrive in distant organs [7, 8]. In this context, the epithelial-to-mesenchymal transition (EMT) could represent the key biological process adopted by epithelial cancer cells to promote tissue dissemination [9]. On note, in our recent study, we demonstrated a putative association between the occurrence of EMT and the development of breast cancer cells showing an osteoblast-like cells phenotype in lesions with microcalcifications [10, 11]. In addition, we observed that the presence of breast osteoblast-like cells (BOLCs) in breast infiltrating cancer was associated with the formation of bone metastatic lesions within 5 years from diagnosis [12, 13].

The main aim of this study was to investigate the putative association among the presence of prostate cancer cells, defined as prostate osteoblast-like cells (POLCs), and showing the expression of typical morphological and molecular characteristics of osteoblasts, the development of bone metastasis within 5years of diagnosis, and the uptake of ^18^F-choline evaluated by PET/CT analysis.

## 2. Materials and Methods

### 2.1. Collection of Prostate Samples

In this study, we enrolled 110 patients undergoing prostate biopsies. From this selection, we collected prostate biopsies from each patient and, when available, data of PET/CT analysis. The study was approved by Institutional Ethical Committee of the “Policlinico Tor Vergata.” Experimental procedures here reported were performed in agreement with the The Code of Ethics of the World Medical Association (Declaration of Helsinki). All patients have signed the informed consent prior to surgical procedures. From each sample, paraffin serial sections were used for both histological and immunohistochemical investigation. Also, 1 mm^3^ of tissue were studied by transmission electron microscopy and microanalytical analysis. Exclusion criteria were history of previously or concomitant other neoplastic diseases, autoimmune diseases, viral chronic infections (HBV, HCV, and HIV), and any antitumoral treatment received before biopsy.

### 2.2. Histology

Fixation and haematoxylin and eosin staining were performed as previously described [14].

### 2.3. Immunohistochemistry

To study the immunophenotypical profile of prostate metastatic cells, we performed immunohistochemical reactions to investigate the expression of the following biomarkers: vimentin (EMT), BMP-2, PTX-3, RUNX2, RANKL, and VDR (mineralization process). For antigen retrieval, 3 *μ*m thick paraffin sections were treated with citrate pH 6.0 or EDTA citrate pH 7.8 buffers (95°C for 30 min). Then, primary antibodies listed in Table 1 were incubated for 1 hour at room temperature. HRP-DAB Detection Kit (UCS Diagnostic, Rome, Italy) was used to reveal the reaction of primary antibodies with their specific target. Immunohistochemical signal was assessed independently by two investigators by counting the number of positive cancer cells (out of a total of 500 in randomly selected regions).

### 2.4. Transmission Electron Microscopy (TEM) and Energy Dispersive X-Ray (EDX) Microanalysis

Small fragment of prostate tissue (1 mm^3^) was fixed in 4% paraformaldehyde and postfixed in 2% osmium tetroxide. Then, the sample was dehydrated in alcohol and infiltrated with propylene oxide before being embedded in Epon (Agar Scientific, Stansted CM24 8GF, Essex, United Kingdom) [15]. Eighty-micrometer ultrathin sections were cut by ultramicrotome and mounted on copper grids. All samples were examined with a transmission electron microscope (Model JEM-1400, JEOL) [16–18].

For EDX microanalysis, 80 *µ*m ultrathin sections were mounted on copper grids. Hydroxyapatite crystals were identified by EDX detector (Thermo Scientific, Waltham, MA, USA) at an acceleration voltage of 75KeV and magnification of 12.000 [16–18]”.

### 2.5. 18F-Choline PET/CT Analysis

Among patients enrolled in the study, 11 were subjected to ^18^F-methylcholine (^18^F-choline) PET/CT analysis. Results of 18F-choline PET/CT were collected to verify a possible correlation between ^18^F-choline uptake in prostate tumours and the presence of POLCs. ^18^F-choline PET/CT analysis was performed as previously described [19, 20]. From each patient, standardized uptake value (SUV) max and SUV average were recorded.

### 2.6. Retrieval and Analysis of Gene Expression Datasets

Gene expression data from two studies in prostate cancer patients [21, 22] were retrieved from the cBioPortal platform. Expression of the selected genes was compared between primary tumours and metastatic CRPC and, for the second dataset, among primary and different metastatic sites. Heatmaps show the results of unsupervised hierarchical clustering based on the gene set expression.

### 2.7. Statistical Analysis

We performed groupwise comparisons of the expression of analysed biomarkers through nonparametric Kruskal–Wallis test (KW) (*p* < 0.05). Post hoc testing was performed by the Mann–Whitney test [12].

## 3. Results

### 3.1. Histological Classification

Prostate biopsies were classified in 44 benign lesions (BL) and 66 malignant lesions according to EAU-ESTRO-SIOG Guidelines 2017 [23]. We subdivided the malignant lesions in those (BM+, *n* = 23) taken from patients with clinical evidence of bone metastasis and those (BM−, *n* = 43) from patients without clinical evidence of bone metastasis after 5 years from diagnosis. From radiological point of view, all metastatic sites showed typical characteristics of osteoblastic lesions. Calcifications were present in 38 out of 110 prostate biopsies. In particular, we observed psammoma bodies in 32% of BL, 87% of BM+, and 79% of BM−. Patient baseline characteristics are reported in Table 2.

### 3.2. EMT Characterization

**Figure 1:**
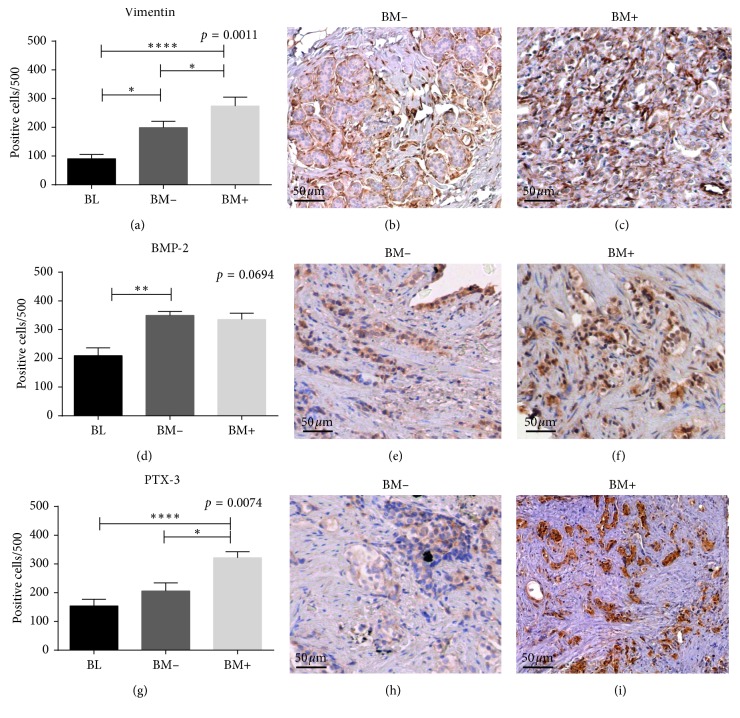
Immunohistochemical analysis of vimentin, BMP-2, and PTX-3. (a) Graph shows the number of vimentin-positive prostate cells in BL, BM+, and BM− lesions. (b) Vimentin-positive prostate cancer cells in BM− lesions (scale bar represents 50 *µ*m). (c) Image shows numerous vimentin-positive prostate cancer cells in BM+ lesions (scale bar represents 50 *µ*m). (d) Graph shows the number of BMP-2-positive prostate cells in BL, BM+, and BM− lesions. (e) BM+ lesion displaying numerous BMP-2-positive cancer cells (scale bar represents 50* µ*m). (f) BMP-2-positive prostate cancer cells in BM+ lesions (scale bar represents 50* µ*m). (g) Graph shows the number of PTX-3-positive prostate cells in BL, BM+, and BM− lesions. (h) Rare PTX-3-positive cells in BM− lesions (scale bar represents 50* µ*m). (i) Image shows several PTX-3-positive prostate cancer cells in BM+ (scale bar represents 50* µ*m).

Immunohistochemical analysis of vimentin expression was performed in order to evaluate the number of prostate cells that acquire mesenchymal phenotype (Figure 1(a)–1(c)). As shown in a recent study [24], significant group effect was detected in the rate of vimentin-positive prostate cells (*p*=0.0025), and post hoc testing showed a significantly higher rate of vimentin-positive prostate cells in BM+ (274.4 ± 30.76) compared to both BL (90.79 ± 14.82) and BM− (198.8 ± 22.8) (BL vs BM− *p*=0.0298; BL vs BM+ *p* < 0.0001; BM− vs BM+ *p*=0.047).

### 3.3. Expression of Bone Markers in Prostate Tissues

Our results showed a significant group effect on BMP-2 expression (*p*=0.0494), and post hoc testing showed increased BMP-2 expression in BM− (349.8 ± 13.13) compared to BL (BL 209.2 ± 39.22) (BL vs BM−*p* = 0.0083) (Figures 1(d)–1(f)). No other significant differences were found (BM+ 335.1 ± 22.05). Our results show that prostate cells, especially cancer cells, express PTX-3, an innate-immune protein. We observed a significant group effect on PTX-3 (*p*=0.0076). Post hoc testing showed very strong expression of PTX-3 in the cytoplasm of BM+ (321.77 ± 21.10) compared to both BL (154.1 ± 23.16) and to BM− (205.8 ± 28.51) (BL vs BM− *p*=0.2205; BL vs BM+ *p* < 0.0001; BM− vs BM+ *p*=0.0158) (Figures 1(g)–1(i)).

### 3.4. Immunophenotypic Characterization of POLCs

**Figure 2:**
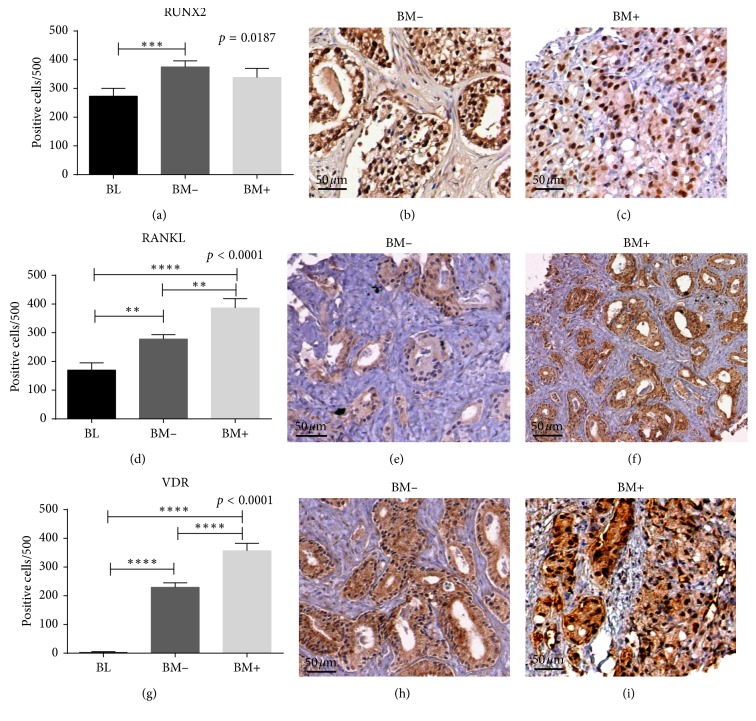
Expression of bone markers in prostate cells. (a) Graph shows the number of RUNX2-positive prostate cells in BL, BM+, and BM− lesions. (b) Numerous nuclear RUNX2-positive cancer cells in BM− lesions (scale bar represents 50* µ*m). (c) Nuclear RUNX″ expression in prostate cancer cells of a BM+ patient (scale bar represents 50* µ*m). (d) Graph displays the number of RANKL-positive prostate cells in BL, BM−, and BM+ lesions. (e) RANKL expression in a case of BM− patient (scale bar represents 50* µ*m). (f) Numerous prostate cancer cells expressing RANKL in BM+ (scale bar represents 50 *µ*m). (g) Graph shows the number of nuclear VDR-positive prostate cells in BL, BM−, and BM+ lesions. (h) VDR-positive prostate cancer cells in a BM− lesion (scale bar represents 50 *µ*m). (i) Several nuclear VDR-positive prostate cancer cells in a BM+ lesion (scale bar represents 50 *µ*m).

A significant group effect was observed on the number of RUNX2-positive cancer cells (*p*=0.0187), after which post hoc testing showed significantly in BM− (375.4 ± 20.97) respect to BL (273.3 ± 27.14) (*p*=0.0006). No other significant differences were found (BM+ 338.2 ± 31.84) (Figures 2(a)–2(c)). RANKL exhibited a significant group effect (*p* < 0.0001), and in post hoc testing, its expression was significantly higher in BM+ (386.7 ± 32.26) with respect to both BL (169.6 ± 25.74) and BM− (278.3 ± 15.24) (BM+ vs BL *p*=0.0011; BM+ vs BM− *p*=0.0014) (Figures 2(d)–2(f)). Also, significant differences were observed by comparing BL and BM− groups (*p* < 0.0001). VDR exhibited a similar group effect (*p* < 0.0001) (Figure 2(g)–2(i)). In addition, we detected significantly higher VDR expression when comparing BM+ (357 ± 25.59) to both BL (3.58 ± 2.03) and BM− (229.5 ± 15.55) (BL vs BM− *p* < 0.0001; BL vs BM+ *p* < 0.0001; BM− vs BM+ *p*=0.0002) (Figure 2(g)). In particular, the signal in BM+ appeared very intense both in nucleus and in cytoplasm (Figure 2(h)), while it was less intense and mainly nuclear in BM− (Figure 2(i)).

### 3.5. Expression of POLC Biomarkers in Gene Expression Datasets

**Figure 3:**
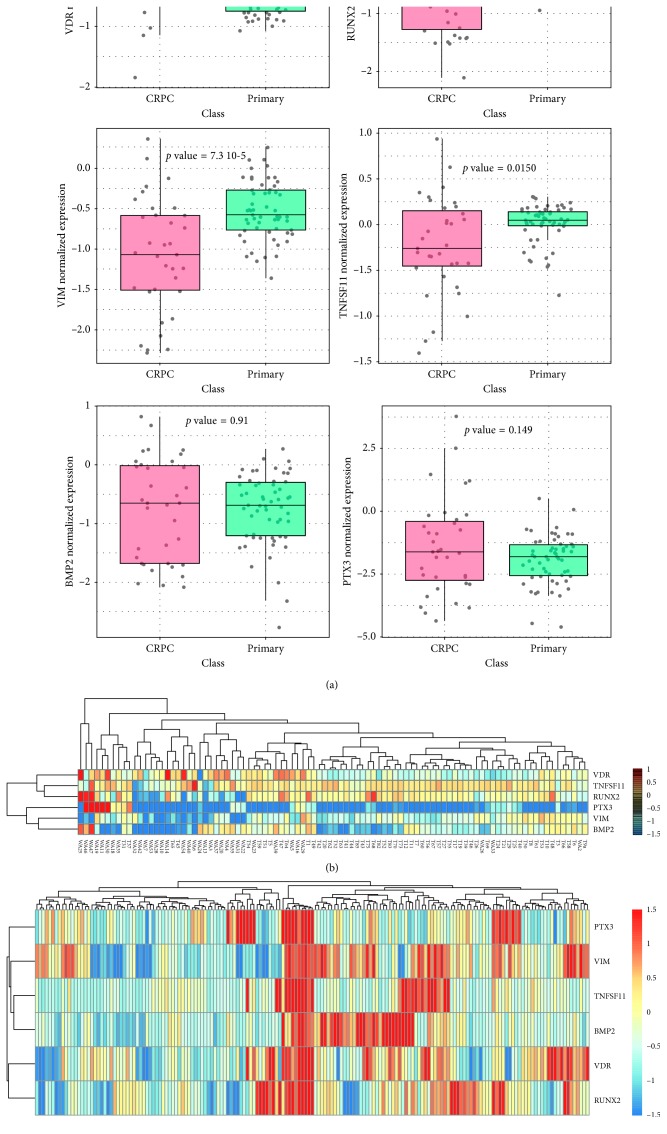
Expression of bone markers in prostate cancer patient datasets. (a) Graphs show the mRNA levels of the genes VDR, RUNX2, vimentin, TNFSF11, BMP-2, and PTX3 in metastatic castration resistant prostate cancer (CRPC) and primary prostate tumours (primary). (b) Unsupervised hierarchical clustering of metastatic (WA) and primary (T) prostate cancers based on expression of the indicated gene set. Metastatic samples are labelled in red; primary samples are labelled in black. (c) Unsupervised hierarchical clustering of primary (prostate) and metastatic prostate cancers at the indicated distinct metastatic sites. Primary/localized samples are indicated in black; distal metastases are indicated in red.

We examined expression of the EMT and bone markers studied by IHC in public datasets comprising gene expression profiling data from patients with primary tumours or metastatic castration-resistant prostate cancer (CRPC). Individual gene comparisons did not show a univocal behaviour (Figure 3(a)). Only VDR expression was significantly upregulated in CRPC compared to primary tumours. Interestingly, however, the small set of genes was able to discriminate most of the primary tumours from metastatic CRPCs in unsupervised clustering (Figure 3(b)). Furthermore, analysis of a second dataset with annotated metastatic sites showed that the gene set expression was remarkably higher in tumour specimens taken from bone metastases compared to primary tumours and other metastatic sites (Figure 3(c)).

### 3.6. Prostate Calcifications

**Figure 4:**
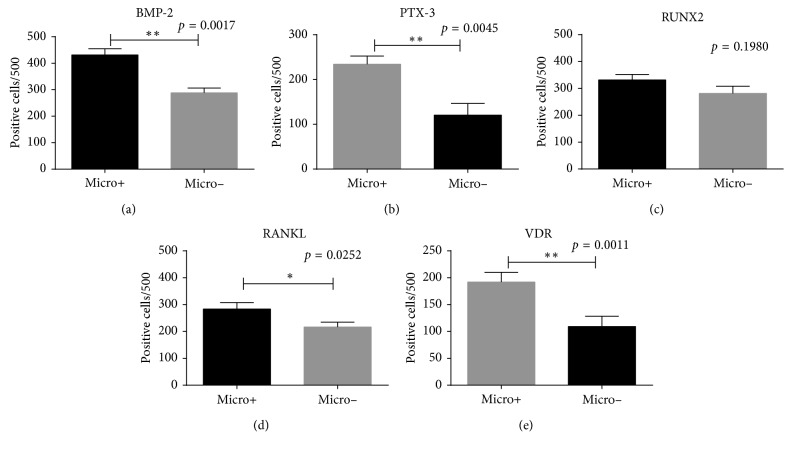
Expression of bone markers in prostate lesions with or without calcification. (a) Graph shows the number of BMP-2-positive prostate cells in Micro+ and Micro− lesions. (b) Graph displays the number of PTX-3-positive prostate cells in Micro− and Micro+ lesions. (c) Graph shows the number of RUNX2-positive prostate cells in Micro+ and Micro−lesions. (d) Graph displays the number of RANKL-positive prostate cells in Micro+ and Micro−lesions. (e) Graph shows the number of VDR-positive prostate cells in Micro+ and Micro− lesions.

In order to verify if the presence of prostate microcalcifications was linked to the expression of mineralization factors, we subdivided our samples in prostate lesions with (Micro+) or without (Micro−) calcifications, independently from the type of lesion. We observed significantly higher expression of BMP-2 in Micro+ respect to Micro− (Micro+ 431.60 ± 23.35 vs Micro− 288.30 ± 18.00; *p*=0.0017) (Figure 4(a)). We found an increase of PTX-3-positive prostate cells in Micro+ as compared to Micro− (Micro+ 234.20 ± 18.40 vs Micro− 120.7 ± 25.82; *p*=0.0045) (Figure 4(b)). Conversely, no significant difference was observed for the analysis of RUNX2-positive prostate cells (Micro+ 331.50±20.52 vs Micro− 281.50 ± 26.65; *p*=0.1980) (Figure 4(c)). Analysis of RANKL showed a significant difference between the presence of RANKL-positive prostate cells between Micro+ and Micro− (Micro+ 283.70 ± 24.23 vs Micro− 216.60 ± 18.07; *p*=0.0252) (Figure 4(d)). Finally, significant differences in VDR expression were observed (Micro+ 192.00 ± 18.02 vs Micro− 109.30 ± 19.14; *p*=0.001) (Figure 4(e)).

### 3.7. Ultrastructural Characterization of Prostate Cancer Cells

**Figure 5:**
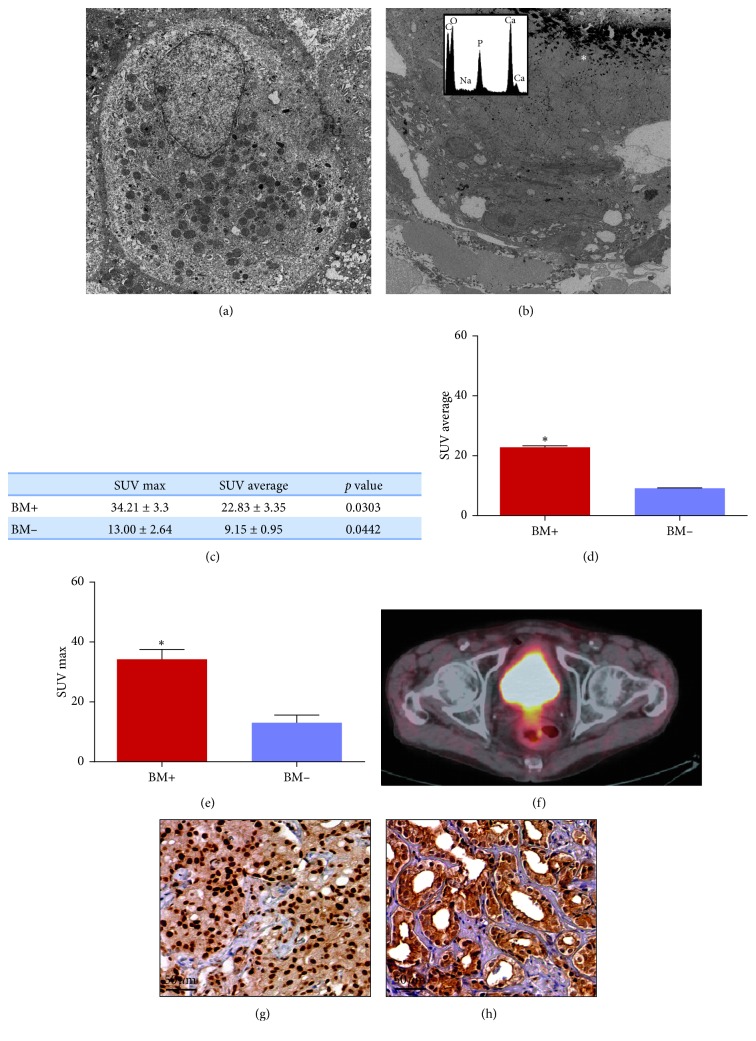
Ultrastructural and molecular imaging analysis. (a) Electron micrograph shows prostate cancer cells of a BM− biopsy. (b) Prostate cancer cells next to calcium-phosphate calcification in a BM+ lesion. SUV max and SUV average of BM+ and BM− lesions. (c) Graph shows significant difference between the SUV max value of BM+ and BM−patients. (d) Graph shows significant difference between the SUV average value of BM+ and BM− patients. (e) Graph shows significant difference between the SUV max value of BM+ and BM− patients. (f) Dual fusion 18F-choline PET/CT image of BM+ patients. (g) Image displays numerous RUNX2-positive prostate cancer cells in BM+ patient of (e) (scale bar represents 50 *µ*m). (h) Image displays numerous RANKL-positive prostate cancer cells in BM+ patient of (e) (scale bar represents 50 *µ*m).

TEM analysis allowed us to characterize ultrastructure of prostate cells in malignant lesions. Specifically, we observed both cuboidal and large spindle-shaped cells with abundant clear cytoplasm in BM+ (Figure 5). Moreover, in these lesions, we identified several calcifications and prostate cancer cells with morphological appearance of osteoblasts containing cytoplasmic electrondense granules made of HA (Figure 5). In addition, EDX microanalysis demonstrated that all calcifications here detected were made of calcium-phosphate (hydroxyapatite) (Figure 5).

### 3.8. 18F-Choline PET/CT Analysis

We collected PET/CT data of 11 patients: 5 BM+ and 6 BM− (Figure 5). Despite the low number of patients, we found significant differences between both SUV max and SUV average between BM+ and BM− (Figure 5, 5). Noteworthy, the cancer lesions with higher value of SUV max (BM+ patients) (Figure 5) were characterized by the presence of calcium-phosphate calcifications and a higher number (>300) of RUNX2-positive (Figure 5) and RANKL-positive (Figure 5) prostate cancer cells.

## 4. Discussion

Prostate metastasis to the bone more often results in osteoblastic lesions, though it is known that prostate bone metastases can display both blastic and lytic characteristics during the early phases of their formation [25]. In addition, there is evidence that during the early phases of osteoblastic metastases formation, it is possible to observe osteolytic lesions, suggesting an overall increase of bone remodeling at these sites. The pathophysiology of bone metastases is frequently explained by the theory of the vicious cycle proposed for the first time by Mundy and Guise [26]. According to this theory, cancer cells resident in bone cause bone destruction because they are capable to stimulate osteoclast activity. In return, cancer cells receive positive feedbacks from humoral factors released by the bone microenvironment during bone destruction and remodeling [27]. Indeed, it is widely accepted that the bone microenvironment is crucial to the success of cancer cells in bone.

In a recent study, we described for the first time the characteristics of prostate cells involved in the production of prostate calcifications demonstrating their similarity with osteoblasts [24]. In addition, our research group described the presence of osteoblast-like cells in breast cancer (BOLCs) showing a correlation between the appearance of BOLCs in primary lesions and development of bone metastases. Based on these studies, the main aim of this study was to investigate the possible correlation between the presence of prostate cancer cells showing expression of typical morphological and molecular markers of osteoblasts and the development of bone metastasis in prostate cancer patients within 5 years from diagnosis of primary lesion. To this end, we collected 110 prostate biopsies (44 benign and 66 malignant lesions). Malignant lesions were subdivided in biopsies from patients with clinical evidence of bone metastasis (BM+, *n* = 23) and those from patients without clinical evidence of bone metastasis (BM−, *n* = 43).

As already reported by Scimeca et al., we found a significant correlation between vimentin expression, one of the most important markers of mesenchymal cells [14], and the presence of prostate osteoblast-like cells (POLCs). Specifically, our data showed a significant increase of positive cells in prostate cancer of BM+ group as compared with BM−. In addition, we proved that primary prostate cancer lesions of BM+ patients were characterized by the expression of osteogenic molecules able to induce osteoblast differentiation and to increase osteoblast function such as mineralization. Among them, BMP-2 is a potent inducer of bone formation through the stimulation of osteoblast differentiation. BMP-2 exerts this effect via two types of serine/threonine kinase receptors: BMP-2 binds the type II receptor, which subsequently activates the type I receptor by a direct association [28]. Our results showed an increase of BMP-2 expression in prostate malignant lesions. Conversely, the absence of significant differences of BMP-2 expression between BM+ and BM− suggests that it could be involved in the early phases of cancer transformation rather than during metastatic process. In support of this, several studies demonstrated the ability of BMP-2 to induce malignant transformation of epithelial tissues [29–31]. However, we also demonstrated the association between BMP-2 expression and the presence of prostate calcifications, regardless of the lesion type. Thus, BMP-2, in association to EMT phenomenon, can participate to induce mesenchymal-like cells to acquire osteoblast phenotype. As concerns PTX-3, PTX-3 is a multifunctional glycoprotein produced by a variety of cells [32, 33]; our results displayed a significant correlation between the presence of PTX-3-positive prostate cells and bone metastasis formation. Also, it is important to emphasize that BM− group showed the same number of PTX-3-positive cells of BL, suggesting that the presence of PTX-3-positive cells could represent a reliable predictive element for the development of bone metastasis from prostate cancer. These data are in line with recent studies that demonstrated the involvement of PTX-3 in osteoblast proliferation, differentiation and function [34–36], and bone metastasis from breast cancer formation.

To further characterize the phenotype of POLCs, we investigate the expression of the main markers of osteoblasts, RUNX2, RANKL, and VDR. RUNX2 is the first transcription factor required for the determination of the osteoblast lineage [37]. In particular, RUNX2 is detected first in preosteoblasts and its expression is upregulated during the early phases of osteoblast differentiation. In line with this, our results displayed an increase of RUNX2-positive prostate cells in malignant lesions respect to BL, but no difference was observed between BM+ and BM−. Therefore, the acquisition of RUNX2 expression by prostate cells seems to be linked to cancer transformation rather than to metastatic process.

In agreement with the physiological role of RUNX2 in osteoblast function [38], we did not observe an increase of RUNX2-positive cells in Micro+ with respect to Micro− lesions. Indeed, mature osteoblasts lose the expression of RUNX2 during the mineralization phase of bone formation. Conversely, analysis of RANKL and VDR showed a putative correlation among the presence of RANKL and/or VDR positive prostate cancer cells, bone metastasis formation, and microcalcifications. As regards the formation of bone metastasis, the presence of RANKL-positive prostate cancer cells can trigger osteoclast activity by binding to the osteoclast receptor RANK [39]. Indeed, RANKL is a type II membrane protein expressed by osteoblasts that is able to induce osteoclasts proliferation and function. In addition, at the primary lesion site, RANKL expression can reflect the presence of cells responsible for microcalcification production. Similar to what occurs during bone mineralization, our data support the hypothesis that the nuclear translocation of VDR participates in production of microcalcifications in prostate lesions. Thus, nuclear translocation of VDR could be considered a marker of POLCs since it could be linked to bone metastasis formation. Notably, nuclear VDR is the only protein that we did not find expressed in BL, among all proteins studied here. This evidence candidates nuclear VDR as a reliable prognostic and/or predictive marker of prostate cancer occurrence. Combined analysis of this set of genes in patients with primary and metastatic prostate cancer further showed that deregulated expression of these markers of EMT, bone mineralization, and osteoblastic differentiation occurred preferentially in the setting of metastatic disease and particularly at metastases in bone, further supporting their relevance as adverse prognostic markers.

It is important to highlight that in this study, POLCs were also characterized from the ultrastructural point of view. In particular, we observed the presence of cytoplasmic vesicles containing HA granules in prostate cancer cells showing osteoblast phenotype (POLCs) [24, 40–42].

Of note, although preliminary, our data showed a significant correlation between the uptake of 18F-choline PET/CT and the presence of POLCs in prostate cancer tissues. If confirmed in a larger patient cohort, this evidence could provide the scientific rationale for the development of algorithms able to predict the metastatic potential of primary prostate cancer lesions by 18F-choline PET/CT analysis [43].

This study proposes a new cell type generated by a process of cell transdifferentiation and related to formation of bone metastasis: the POLCs. Although our data require further investigations about the molecular mechanisms of both POLCs generation and metastasization to the bone, this study opens new and interesting prospective for the management of prostate cancer patients. The presence of POLCs could become prognostic markers for occurrence of bone metastatic disease.

## 5. Conclusion

The clinical course of metastatic bone disease in prostate cancers is often long, with patients experiencing sequential skeletal complications over a period of several years. These include bone pain, fractures, hypercalcemia, and spinal cord compression, all of which may profoundly impair patient's quality of life. In addition, once prostate tumour cells are engrafted in the skeleton, curative therapy is no longer possible and palliative treatment becomes the only option. Thus, the identification of early markers of bone metastasis and especially the characterization of the cells involved in the metastatic process can lay the foundation for the identification of new tools for monitoring, prevention, or cure of bone metastatic diseases and providing support to the physicians in the management of prostate patients. In this context, positron emission tomography (PET)/computed tomography (CT) has emerged as a significant and promising staging modality for primary, recurrent, and metastatic prostate cancer. Much more important, the identification of highly sensitive and specific radiotracers can implement the therapeutic/diagnostic perspectives for prostate cancer patients “opening the way” for the development of new theranostic approaches. PSMA PET/CT ligands labelled with ^18^F and ^68^Ga have certainly revolutionized the management of metastatic prostate cancer selecting patients who may benefit from targeted systemic radionuclide therapy. In a nuclear oncology theranostic design, ^68^Ga-PSMA already constitutes the diagnostic positron-emitting of beta^−^ emitter Lutetium-177 PSMA (^177^Lu-PSMA) [44] and alpha-emitter Actinium-225 PSMA (^225^Ac-PSMA) [45]. Finally, the results reported here about the phenotypic characterization of POLCs could provide a scientific rationale for the development of theranostic anti-POLC radiomolecules for the cure and prevention of prostate cancer bone metastasis.

## Figures and Tables

**Table 1:** List of primary antibodies.

Antibody	Characteristics	Dilution	Retrieval
Antivimentin	Mouse monoclonal clone V9; Ventana, Tucson, AZ, USA	Prediluted	EDTA citrate pH 7.8
Anti-BMP-2	Rabbit monoclonal clone N/A; Novus Biologicals, Littleton, CO, USA	1 : 250	Citrate pH 6.0
Anti-PTX-3	Rat monoclonal clone MNB1; AbCam, Cambridge, UK	1 : 100	Citrate pH 6.0
Anti-RUNX2	Mouse monoclonal clone EPR14334; AbCam, Cambridge, UK	1 : 100	Citrate pH 6.0
Anti-RANKL	Rabbit monoclonal clone 12A668; AbCam, Cambridge, UK	1 : 100	EDTA citrate pH 7.8
Anti-VDR	Rabbit polyclonal clone NBP1-19478; Novus Biologicals, Littleton, CO, USA	1 : 100	Citrate pH 6.0

**Table 2:** Baseline characteristics of patients.

	n	Age ≤55	Age ≥55	Gleason ≤6	Gleason 7	Gleason ≥8	PSA (ng ml^–1^)
BL	44	20	24	/	/	/	/
BM+	23	5	18	4	4	15	1122.11 ± 1348.02
BM−	43	13	30	13	6	25	1001.09 ± 147938

## Data Availability

The data used to support the findings of this study are included within the article. Gene expression data from two studies in prostate cancer patients (reference [21] and [22]) were retrieved from the cBioPortal platform. Expression of the selected genes was compared between primary tumours and metastatic CRPC and, for the second dataset, among primary and different metastatic sites.
