# Erratum to “Prostate Osteoblast-Like Cells: A Reliable Prognostic Marker of Bone Metastasis in Prostate Cancer Patients”

**DOI:** 10.1155/2019/7843735

**Published:** 2019-03-11

**Authors:** Manuel Scimeca, Nicoletta Urbano, Rita Bonfiglio, Sarah Natalia Mapelli, Carlo Vittorio Catapano, Giuseppina Maria Carbone, Sara Ciuffa, Mario Tavolozza, Orazio Schillaci, Alessandro Mauriello, Elena Bonanno

**Affiliations:** ^1^Department of Biomedicine and Prevention, University of Rome “Tor Vergata”, Via Montpellier 1, Rome 00133, Italy; ^2^University San Raffaele, Via di Val Cannuta 247, Rome 00166, Italy; ^3^Nuclear Medicine, Policlinico “Tor Vergata”, Rome, Italy; ^4^Department of Experimental Medicine and Surgery, University “Tor Vergata”, Via Montpellier 1, Rome 00133, Italy; ^5^Università della Svizzera Italiana (USI), Institute of Oncology Research (IOR), Via Vela 6, Bellinzona, Switzerland; ^6^IRCCS Neuromed, Pozzilli, IS, Italy; ^7^IRCCS Neuromed Lab. “Diagnostica Medica” and “Villa Dei Platani”, Avellino, Italy

In the article titled “Prostate Osteoblast-Like Cells: A Reliable Prognostic Marker of Bone Metastasis in Prostate Cancer Patients” [1], the name of the third author was reversed. The corrected authors' list is shown above.
