# Correction to “Prostate Osteoblast‐Like Cells: A Reliable Prognostic Marker of Bone Metastasis in Prostate Cancer Patients”

**DOI:** 10.1155/cmmi/9789206

**Published:** 2025-12-30

M. Scimeca, N. Urbano, R. Bonfiglio, S. N. Mapelli, C. V. Catapano, G. M. Carbone, S. Ciuffa, M. Tavolozza, O. Schillaci, A. Mauriello, and E. Bonanno, “Prostate Osteoblast‐Like Cells: A Reliable Prognostic Marker of Bone Metastasis in Prostate Cancer Patients,” *Contrast Media & Molecular Imaging* 2018, no. 1 (2018): 1–12, https://doi.org/10.1155/2018/9840962.

In the article titled “Prostate Osteoblast‐Like Cells: A Reliable Prognostic Marker of Bone Metastasis in Prostate Cancer Patients,” there were some errors in Figure 2.

More specifically, the image of VDR‐positive prostate cancer cells in Figure 2h is incorrect, while the scale bars in Figure 2e,f should state 250 µm.

These errors have occurred due to an oversight by the authors during figure assembly, and Figure 2 should be corrected as follows:

**Figure 2:**
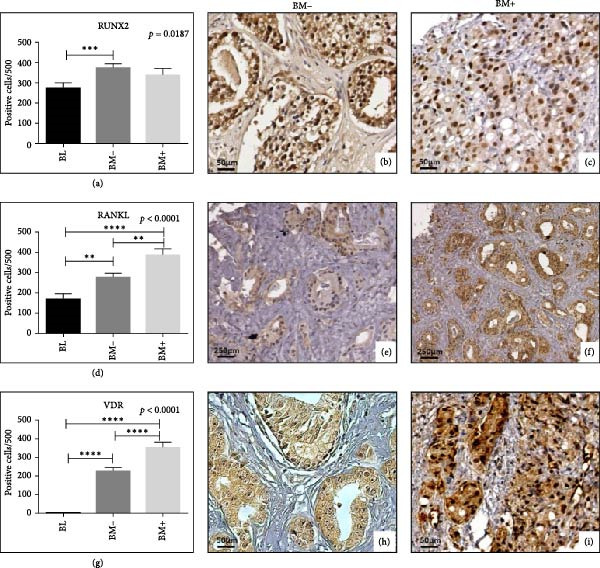
Expression of bone markers in prostate cells. (a) Graph shows the number of RUNX2‐positive prostate cells in BL, BM+, and BM− lesions. (b) Numerous nuclear RUNX2‐positive cancer cells in BM− lesions (scale bar represents 50 µm). (c) Nuclear RUNX″ expression in prostate cancer cells of a BM+ patient (scale bar represents 50 µm). (d) Graph displays the number of RANKL‐positive prostate cells in BL, BM−, and BM+ lesions. (e) RANKL expression in a case of BM− patient (scale bar represents 250 µm). (f) Numerous prostate cancer cells expressing RANKL in BM+ (scale bar represents 250 µm). (g) Graph shows the number of nuclear VDR‐positive prostate cells in BL, BM−, and BM+ lesions. (h) VDR‐positive prostate cancer cells in a BM− lesion (scale bar represents 50 µm). (i) Several nuclear VDR‐positive prostate cancer cells in a BM+ lesion (scale bar represents 50 µm).

We apologize for these errors.

